# Subcutaneous Purgation

**Published:** 1912-12

**Authors:** 


### ADDITIONAL ABSTRACTS

SUBCUTANEOUS PURGATION. Dr. Paul Barnot, *Paris Medicale,* June 29, 1912. The indications are: 1. Anything preventing the use of the mouth, as incessant vomiting, lead colic, voluntary poisoning—when both apomorphine and purgatives may be administered hypodermatically—,coma, etc. 2. Requirement of too small doses to be efficient by mouth or contraindication to local irritation, as in gastric ulcer, appendicitis, gastro-enteric neoplasm. 3. Need of continuous action, as when the digestive tract has been exhausted by laxatives or by anaesthesia.

He prefers sulphate of magnesium 2—20 cc. of 1% solution but for a more marked action, 1--3cc. of a 10% solution may be used. In obstipation affecting the colon, peristaltic hormone, cascarin, senna, etc., are preferable. Generally speaking, salines are better absorbed and produce less local oedma if approximately of physiologic strength (isotonic). Zulzer and Dohrn's peristaltic hormone solution is employed in a dose of 10—20 cc. subcutaneously or even intravenously. Senna is used as an infusion, sterilized in an autoclave, 1cc. of 5% solution or 5cc. of 1% solution. Ext. rhamni purshianae may be similarly employed. Phenolphthalein is used with a small amount of sodium carbonate.

